# Differential Characteristics of Viral siRNAs between Leaves and Roots of Wheat Plants Naturally Infected with Wheat Yellow Mosaic Virus, a Soil-Borne Virus

**DOI:** 10.3389/fmicb.2017.01802

**Published:** 2017-09-20

**Authors:** Linying Li, Ida Bagus Andika, Yu Xu, Yan Zhang, Xiangqi Xin, Lifeng Hu, Zongtao Sun, Gaojie Hong, Yang Chen, Fei Yan, Jian Yang, Junmin Li, Jianping Chen

**Affiliations:** ^1^College of Plant Protection, Nanjing Agricultural University Nanjing, China; ^2^The State Key Laboratory Breeding Base for Sustainable Control of Pest and Disease, Zhejiang Academy of Agricultural Sciences Hangzhou, China; ^3^Key Laboratory of Biotechnology in Plant Protection of Ministry of Agriculture of China and Zhejiang Province, Institute of Virology and Biotechnology, Zhejiang Academy of Agricultural Sciences Hangzhou, China; ^4^Group of Plant-Microbe Interactions, Institute of Plant Science and Resources, Okayama University Kurashiki, Japan; ^5^Institute of Plant Protection, Shandong Academy of Agricultural Sciences Jinan, China

**Keywords:** soil-borne plant viruses, wheat yellow mosaic virus, viral small interfering RNA, antiviral RNA silencing, deep sequencing

## Abstract

RNA silencing is an important innate antiviral defense in plants. Soil-borne plant viruses naturally infect roots via soil-inhabiting vectors, but it is unclear how antiviral RNA silencing responds to virus infection in this particular tissue. In this study, viral small interfering RNA (siRNA) profiles from leaves and roots of wheat plants naturally infected with a soil-borne virus, wheat yellow mosaic virus (WYMV, genus *Bymovirus*), were analyzed by deep sequencing. WYMV siRNAs were much more abundant in roots than leaves, which was positively correlated with the accumulation of viral RNA. WYMV siRNAs in leaves and roots were predominantly 21- and 22-nt long and equally derived from the positive- and negative-strands of the viral genome. WYMV siRNAs from leaves and roots differed in distribution pattern along the viral genome. Interestingly, compared to siRNAs from leaves (and most other reports), those from roots obviously had a lower A/U bias at the 5′-terminal nucleotide. Moreover, the expression of Dicer-like genes upon WYMV infection were differently regulated between leaves and roots. Our data suggest that RNA silencing in roots may operate differently than in leaves against soil-borne virus invasion.

## Introduction

Virus infection in plants is usually associated with the accumulation of virus-derived small interfering RNAs (vsiRNAs) that play essential roles in antiviral RNA silencing defense by degrading viral RNA in a sequence-specific manner (Llave, 2010; Zhang et al., 2015; Li M.L. et al., 2016). In the antiviral RNA silencing pathway, viral-derived double-stranded RNA (dsRNA) is processed by a ribonuclease III-like protein called Dicer or Dicer-like (DCL), to produce 21- to 24-nucleotide (nt) siRNAs. These are then incorporated into RNA-induced silencing complexes (RISCs) containing Argonaute (AGO) to mediate sequence-specific viral RNA degradation (Aliyari and Ding, 2009; Bologna and Voinnet, 2014). High-throughput sequencing and comprehensive analysis of vsiRNAs in infected plant samples have expanded our knowledge of the biogenesis of vsiRNAs and their participation in the regulation of host gene expression and virus–host interactions as well as in shaping the evolution of viruses (Llave, 2010; Zhu and Guo, 2012; Kutnjak et al., 2015; Hadidi et al., 2016). In particular, recent comparative studies of siRNAs of the same virus in different host plants (genotypes), or siRNAs of different viruses (strains) in the same host plant, have provided deeper insights into the link between viral pathogenicity and vsiRNA profiles (Naveed et al., 2014; Margaria et al., 2015, 2016; Ogwok et al., 2016). Other reports also suggest that the biogenesis of vsiRNAs of plant viruses differs in their plant hosts from that in their insect vectors, for example, with tomato spotted wilt virus (genus *Tospovirus*) in the host *Arachis hypogaea* and the vector *Frankliniella fusca* (Fletcher et al., 2016), and with rice stripe virus (genus *Tenuivirus*) in the hosts *Oryza sativa* or *Nicotiana benthamiana* and the vector *Laodelphax striatellus* (Xu et al., 2012). Nevertheless, most studies of vsiRNA profiles in plants have used infected leaf tissue, and there is little evidence from other tissues. In our recent study of cucumber green mottle mosaic virus (genus *Tobamovirus*) siRNAs from leaves and fruits, vsiRNAs in leaves were predominantly derived from the viral positive-strand RNA, whereas those in fruits were derived equally from both strands, suggesting that the biogenesis of vsiRNAs might differ in the different tissues (Li J. et al., 2016).

Most plant viruses are transmitted to leaves by arthropod vectors, but some soil-inhabiting organisms, such as plasmodiophorids and nematodes can transmit viruses to plant roots (Hull, 2013). Thus, successful multiplication in plant roots is important for the life cycle of soil-borne viruses. There are few studies of vsiRNA profiles in root tissues and none that have analyzed vsiRNAs in roots from the viruses that infect the plants through soil-inhabiting vectors (Andika et al., 2016). Given the large physiological differences between the underground root and the aerial parts of plants, it is possible that antiviral defense might work differently in plant roots than in leaves and shoots. Since DCL proteins are the primary key enzymes in the RNA silencing pathway, investigation of vsiRNA profiles in roots may provide new insights into the antiviral RNA silencing mechanism against soil-borne virus invasion.

Wheat yellow mosaic virus (WYMV, genus *Bymovirus* and family *Potyviridae*) is an economically important pathogen of winter wheat (*Triticum aestivum*), causing serious yield losses in Japan and China (Namba et al., 1998; Han et al., 2000). WYMV is transmitted by *Polymyxa graminis*, an obligate soil-inhabiting fungus-like protist (Kanyuka et al., 2003). Like other viruses in the genus *Bymovirus*, the WYMV genome consists of two positive single-stranded RNAs (RNA1 and RNA2). RNA1 encodes a large polyprotein that is proteolytically processed into eight proteins; P3, 7k, cylindrical inclusion protein (CI), 14k, genome-linked viral protein (VPg), nuclear inclusion protein a-proteinase (NIa-Pro), nuclear inclusion protein b (Nib), and coat protein (CP). RNA2 encodes a polyprotein processed into a 28-kDa protein (P1) and a 73-kDa protein (P2) (Namba et al., 1998; Adams et al., 2005). P2 is unique to bymoviruses; it is essential for the transmission vectored by *P. graminis* and plays a role in the formation of membranous compartments associated with genome replication of WYMV (Dessens and Meyer, 1996; Adams et al., 2001; Sun et al., 2014). In the monopartite viruses within the family *Potyviridae*, the helper component protease (HC-Pro), the P1 protein, or P1N-PISPO function as RNA silencing suppressors, but the silencing suppressor of bymoviruses has not been identified (Valli et al., 2006, 2017; Tatineni et al., 2012; Mingot et al., 2016; Untiveros et al., 2016). The N-terminal region of P1 of bymoviruses including WYMV, barley yellow mosaic virus (Kashiwazaki et al., 1991) and barley mild mosaic virus (Kashiwazaki, 1996) show significant amino acid homologies with the active domains of the HC-Pro of monopartite potyviruses such as potato virus Y, plum pox virus, tobacco etch virus and tobacco vein mottling virus (Namba et al., 1998). Nonetheless, the roles of P1 in the multiplication and life cycle of bymoviruses need further investigation.

In the present study, the profiles of WYMV vsiRNA derived from leaves and roots of infected wheat plants were comprehensively characterized by deep sequencing. In addition, the expressions of *DCL* and *AGO* gene transcripts in WYMV-infected leaves and roots were analyzed by quantitative PCR.

## Materials and Methods

### Sample Collection and Total RNA Extraction

WYMV-infected plant samples were obtained from a wheat nursery in Linyi, Shandong Province of China, where it was continually used to screen WYMV resistance wheat cultivars during 2012–2016. Wheat plants (cultivar Linmai4) with fully developed stem and jointing as well as having typical yellow mosaic symptoms were collected from the diseased nursery in March, 2016. In parallel, virus-free plants of the same cultivar were obtained from an adjacent field (less than 10 m distance) as a control. Three independent replicates of virus-free and WYMV-infected root and leaves samples were used for the experiment. Roots of each wheat plant (50 mg) were cut into small pieces and ground to a fine powder under liquid nitrogen. Total RNA was extracted using Trizol reagent (Invitrogen, CA, United States) according to the manufacturer’s instructions. The integrity and quality of RNA samples were evaluated by denaturing agarose gel electrophoresis and 2100 Bioanalyzer (Agilent, United States). The presence of WYMV in the roots was confirmed for all samples with a One Step RT-PCR Kit (TOYOBO, Japan) using WYMV-specific primers (W-F, 5′-CAAGGTTGAGGCAGATCGTG-3′; W-R: 5′-CAGATGCGCCGTGTTTCATA-3′).

### Small RNA Sequencing and Bioinformatics Analysis

About 5 μg of total RNA was extracted from each of the leaves and roots of virus-free and WYMV-infected plants for the preparation of small RNA (sRNA) libraries using the Illumina TruSeq Small RNA Sample Preparation Kit (Illumina, United States), while the remaining RNA was used for reverse transcription-quantitative PCR (RT-qPCR) analysis. sRNA high-throughput sequencing was carried out on an Illumina HiSeq 2500 at LC-BIO (Hangzhou, China). Preliminary treatment of raw data was performed as described previously (Li et al., 2013). Briefly, after removal of the 3′ adaptor, low quality and junk sequences using the FASTX-Toolkit^1^, sRNAs with length of 18- to 30-nt were extracted and collapsed for further bioinformatics analysis. To identify WYMV-derived siRNAs, processed reads derived from both leaves and roots of infected and virus-free wheat libraries were mapped to the WYMV genome (NCBI accession No.: PRJNA15358) using Bowtie software^2^ allowing for one mismatch. To facilitate the comparisons between different sized libraries, identified vsiRNA raw reads counts were scaled to “Reads Per Million” (RPM) based on the total sRNA read numbers of the corresponding library. Downstream analyses for the vsiRNAs were carried out using custom perl scripts and Linux bash scripts.

### Reverse Transcription-Quantitative PCR

Equal quantities of total RNA (100 ng) from leaves and roots of virus-free and WYMV-infected plants were used for cDNA synthesis. The first-strand cDNAs were generated from the extracted total RNAs using the Fast Quant RT Kit (Tiangen, Beijing, China) and RT-qPCR was performed on the ABI 7900HT (Applied Biosystems). Primer sets specific for WYMV (WYMV-CP), wheat *AGO1* (TaAGO1), *AGO2* (TaAGO2), *AGO4* (TaAGO4), *DCL2* (TaDCL2), and *DCL4* (TaDCL4) were used for RT-qPCR and ubiquitin (TaU) was used as a reference gene. All the primers used for RT-qPCR were listed in Supplementary Table S1. The RT-qPCR reaction was run in a final volume of 20 μl containing 10 μl of PCR buffer, 0.6 μl of each primer (10 μM/l), 1 μl of template cDNA, and 7.8 μl of DEPC H_2_O. The reaction conditions were: 94°C for 3 min, followed by 40 cycles of 94°C for 20 s, 58°C for 20 s, and 72°C for 20 s.

### Statistical Analysis

One-way ANOVA was performed in this study using Originpro 8.5 and values of *P* < 0.01 were considered significantly different between the samples.

## Results and Discussion

### Overview of WYMV-Derived siRNAs

**FIGURE 1:**
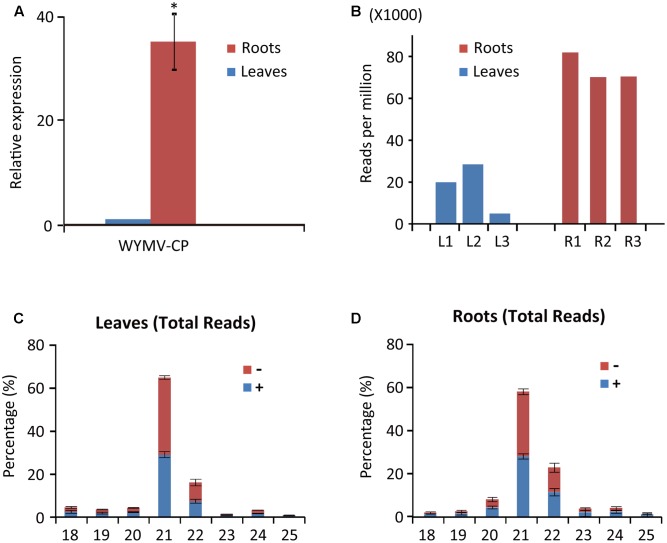
Abundance of WYMV RNA and siRNAs in leaves and roots of wheat plants. **(A)** RT-qPCR analysis of WYMV RNA accumulation. **(B)** Abundance of WYMV siRNAs. **(C,D)** Size distribution of WYMV siRNAs. “-” and “+” indicate siRNAs derived respectively from the complementary (negative) or positive viral genomic strands. Asterisk indicates significant difference at *P* < 0.01 (one-way ANOVA).

Deep sequencing yielded 5,214,377 (838,479 unique), 5,035,946 (1,388,780 unique), and 4,659,474 (1,071,118 unique) total small reads (18–30 nt) from the three leaf libraries, and 9,507,299 (1,714,971 unique), 10,021,025 (2,188,254 unique), and 11,227,881 (2,245,279 unique) total small reads (18–30 nt) from the three root libraries. Large numbers of sRNA reads from both leaf and root libraries were mapped to the WYMV genome (**Table 1** and **Supplementary File S1**), whereas only a very small number (<100 reads) were obtained from the virus-free samples (data not shown). The total and unique vsiRNAs respectively accounted for 0.49–2.85 and 1.14–4.21% of the total sRNAs in leaves, while the corresponding values for root samples were 7.02–8.19 and 4.20–5.17% (**Table 1**). Sequencing results indicated that vsiRNAs were much more abundant in roots than in leaves (**Table 1** and **Figure 1B**). Our RT-qPCR showed that accumulation of WYMV was about 30-fold higher in roots than in leaves (**Figure 1A**), which was positively correlated with the accumulation of vsiRNAs. This result suggests that the high levels of viral replication might provide more dsRNA substrates for the generation of vsiRNAs in roots by the host RNA silencing machinery. Previous studies have also shown that some soil-borne viruses accumulate to a higher level in roots than leaves (Andika et al., 2005, 2013; Gosalvez-Bernal et al., 2008). In contrast to our results with WYMV, two other soil-borne viruses, beet necrotic yellow vein virus (BNYVV, genus *Benyvirus*) and Chinese wheat mosaic virus (genus *Furovirus*) accumulate to higher levels in roots than leaves of *N. benthamiana* plants, but these higher accumulations are not associated with more abundant vsiRNAs in roots (Andika et al., 2005, 2013). Nevertheless, in those studies the viruses were inoculated into the plants by mechanical rub-inoculation of the leaves. Thus, antiviral RNA silencing may respond differently depending on the route of entry of virus into the plant or the viral transmission method. The positive correlation between virus accumulation levels and the abundance of WYMV siRNAs in roots may suggest that initially plant antiviral silencing strongly responds to WYMV multiplication in roots by actively processing viral RNAs into siRNAs, but this antiviral response seems to be ineffective in limiting viral accumulation in roots. As suggested previously (Andika et al., 2016), some soil-borne viruses may be more adapted to roots than aerial tissue for their efficient transmission and multiplication. The suppression activities of RNA silencing suppressors encoded by BNYVV and tobacco rattle virus (genus *Tobravirus*) are more effective in roots than leaves (Andika et al., 2012). It is possible that like other soil-borne viruses, WYMV suppresses antiviral RNA silencing more effectively in roots than in the aerial parts of the plant.

**Table 1:** The read number of small RNAs (sRNAs) and WYMV-derived small interfering RNAs (vsiRNAs) from virus-infected wheat plants.

	Leaves			Roots		
		
	Replicate 1	Replicate 2	Replicate 3	Replicate 1	Replicate 2	Replicate 3
	L1	L2	L3	R1	R2	R3
sRNAs (total)	5,214,377	5,035,946	4,659,474	9,507,299	10,021,025	11,227,881
vsiRNAs (total)^∗^	103,581	143,327	22,997	778,744	702,980	791,399
vsiRNA/sRNAs (total)	1.99%	2.85%	0.49%	8.19%	7.02%	7.05%
sRNAs (unique)	838,479	1,388,780	1,071,118	1,714,971	2,188,254	2,245,279
vsiRNAs (unique)	35,336	36,838	12,206	88,686	91,937	97,287
vsiRNAs/sRNAs (unique)	4.21%	2.65%	1.14%	5.17%	4.20%	4.33%


*^∗^Raw reads which were mapped to the WYMV genome allowing for one mismatch.*

WYMV siRNA populations derived from leaves and roots were predominantly 21- and 22-nt long (**Figures 1C,D**), suggesting they were the products of DCL4 and DCL2 (Deleris et al., 2006; Diaz-Pendon et al., 2007). WYMV siRNAs from both leaves and roots had approximately equal proportions of sense and antisense sequences (**Figures 1C,D**), consistent with the view that viral dsRNA replication intermediates are the main substrates for vsiRNA production (Ahlquist, 2002; Ding, 2010).

### Differential vsiRNA Distribution Patterns between Leaves and Roots along the Viral Genome

**FIGURE 2:**
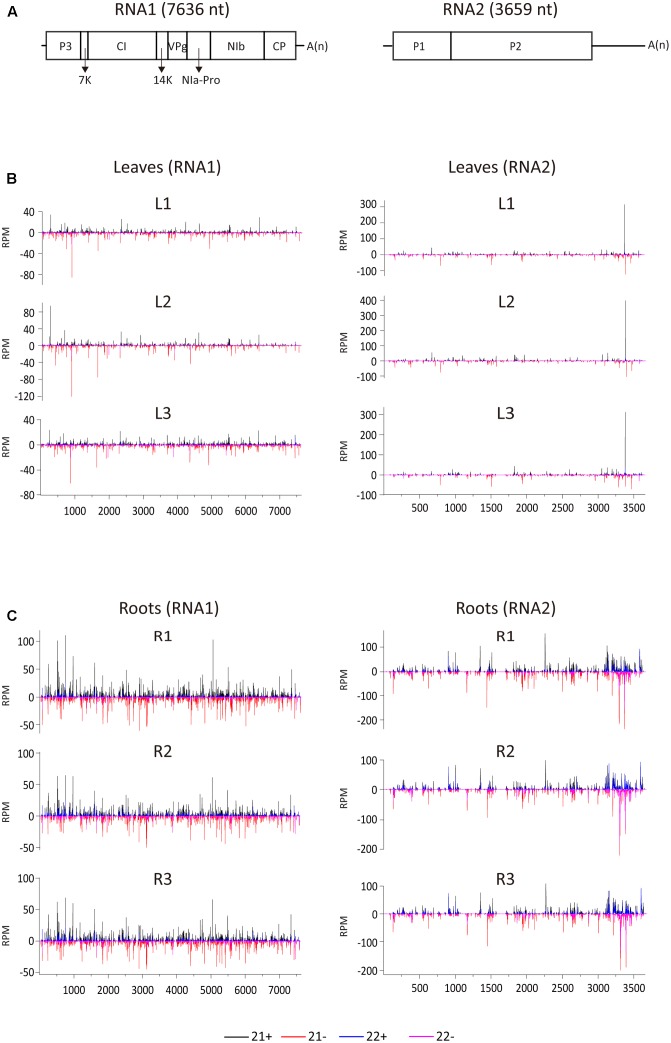
Distribution of WYMV siRNAs (21 and 22 nt) along the viral genome. **(A)** A schematic presentation of the WYMV genome. Boxes indicate the open reading frame for the polyproteins. P3, P3 protein; 7k, 7 kDa protein; CI, cylindrical inclusion protein; 14k, 14 kDa protein; VPg, genome-linked viral protein; NIa-Pro, nuclear inclusion protein a-proteinase; Nib, nuclear inclusion protein b; CP, coat protein; A(n), polyA. **(B,C)** Distribution of WYMV siRNAs (21 and 22 nt) from leaf and root samples (three replicates of each) along the WYMV genome. Color coding indicates vsiRNAs derived, respectively, from the positive (+) and negative viral genomic strands (–). All siRNA reads in this analysis were redundant and normalized.

To examine the vsiRNA distribution pattern, 21- and 22-nt vsiRNAs were aligned to the WYMV genome. Although total vsiRNA numbers differed between the three biological replicates, the vsiRNA distribution patterns were similar within each leaf and root samples, confirming the reliability of the data (**Figure 2**). The 21- and 22-nt siRNAs were distributed along the entire RNA1 and RNA2 sequences including the untranslated regions (UTRs) (**Figure 2**). It was particularly noticeable in RNA2 segment of root samples, vsiRNAs were more densely mapped to 3′-UTR, suggesting that this region is preferentially targeted by DCL for vsiRNA biogenesis (**Figure 2C**). Multiple vsiRNA hotspots were identified in both RNA segments, but the position of those hotpots differed between leaf and root samples. Interestingly, in leaf (but not in root) samples, a very prominent hotspot for a single sense strand 21-nt vsiRNA occurred in the 3′-UTR region of RNA2 (nt position 3379–3399), while in root samples, hotspots composed of several negative strand vsiRNAs were observed in the similar region of 3′-UTR region of RNA2 (**Figures 2B,C**). Highly structured single-stranded viral RNA potentially responsible for vsiRNA hotspots generation as demonstrated previously (Molnar et al., 2005). However, examination of this region using RNAfold^3^ showed no clear relationship between the hotspot and predicted secondary structure (data not shown). Furthermore, three vsiRNA libraries (replicates) were mixed, and unique 21- and 22-nt vsiRNAs were extracted and analyzed. Leaf and root libraries shared substantial numbers of common vsiRNAs, but root libraries had many more tissue-specific vsiRNAs than leaf libraries (**Supplementary Figure S1**), indicating that the higher abundance of vsiRNAs in roots reflects more diverse Dicer cleavage sites.

### A/U Bias at the 5′-Terminal Nucleotide of vsiRNAs Was Higher in Leaves Than Roots

**FIGURE 3:**
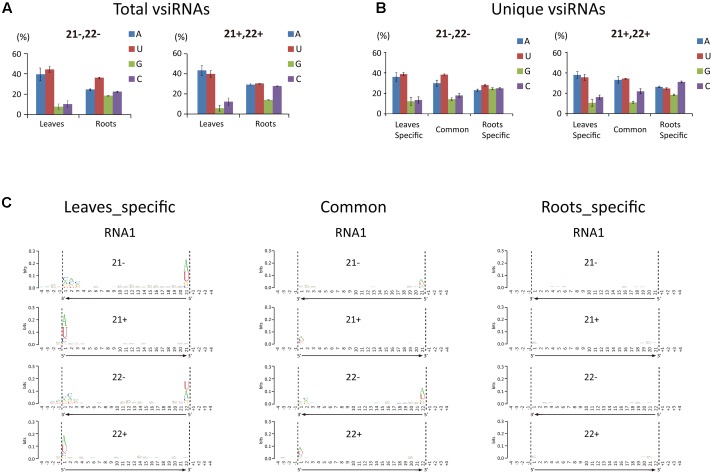
5′-Terminal nucleotide profile of WYMV siRNAs. **(A,B)** Distribution patterns of the 5′-terminal nucleotide of WYMV siRNAs. **(C)** Sequence logo analysis of WYMV siRNAs. 21- and 22-nt WYMV siRNAs corresponding to RNA1 were separately analyzed, and the 4 nt proximal to the 5′ and 3′ ends of the siRNAs in the viral genome sequence were included in the analysis. The overall height of the stack indicates the sequence conservation at that position, while the height of characters within the stack indicates the relative frequency of nucleotide at that position.

The 5′-terminal nucleotide of sRNAs is important in their preferential recruitment by AGO complexes (Mi et al., 2008). Nucleotide bias toward adenine (A) or uracil (U) at the 5′-terminal is typical of vsiRNAs from various organisms including plants, fungi, and insects (Yan et al., 2010; Xu et al., 2012; Li et al., 2013; Mitter et al., 2013; Yang et al., 2014; Margaria et al., 2015, 2016; Yaegashi et al., 2016). Interestingly, our analysis showed that the A/U bias at the 5′-terminal nucleotide of 21- and 22-nt vsiRNA was markedly higher in leaf libraries than in root libraries (**Figure 3A**). Analysis of the unique vsiRNAs showed that leaf-specific and common vsiRNAs have a typical strong A/U preference, whereas root-specific vsiRNAs have no clear preference for the 5′-terminal nucleotide (**Figure 3B**). Sequence logo analysis^4^ (Crooks et al., 2004) was then performed on 21- and 22-nt vsiRNA (unique reads) including the 4 nt proximal to the 5′ and 3′ ends of vsiRNA in the viral genome sequence to identify any sequence conservation within vsiRNAs and in the region surrounding their cleavage sites. No particular nucleotide conservation was found within the internal sequences of vsiRNAs or the surrounding cleavage sites, but the analysis confirmed that the 5′-terminal nucleotide of leaf-specific and to a lesser degree, common vsiRNAs (both polarities) were most frequently A or U, whereas no such preference was observed in root-specific vsiRNAs (**Figure 3C** and **Supplementary Figure S2**).

In *Arabidopsis thaliana*, sRNAs with a 5′-terminal A are preferentially recruited by AGO2 and AGO4, while AGO1 favors sRNAs with a 5′-terminal U (Montgomery et al., 2008; Takeda et al., 2008). The finding that vsiRNAs in plants typically have a strong 5′-terminal bias toward A/U is therefore consistent with the antiviral role of AGO1, AGO2, and AGO4 (Carbonell and Carrington, 2015). In our experiments, the proportion of vsiRNAs with a 5′-terminal A or U was markedly less in roots than leaves (**Figures 3A,B**). Thus although WYMV siRNAs were much more abundant in roots than leaves, it is possible that in roots only a small proportion of WYMV siRNAs are incorporated into RISCs and therefore that antiviral RNA silencing operates less efficiently against WYMV in roots. The reason for the differential 5′-terminal nucleotide profiles of WYMV siRNAs between roots and leaves remains unclear. It is possible that WYMV replication or WYMV-encoded protein(s) alter the normal DCL preferential cleavage sites for vsiRNA production in roots.

### Expression of Dicer-Like Genes Were Differently Regulated between Leaves and Roots during WYMV Infection

**FIGURE 4:**
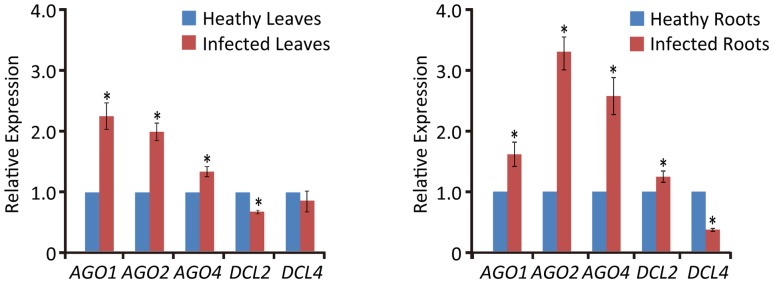
Relative transcript expressions of wheat *AGO1*, *AGO2*, *AGO4*, *DCL2*, and *DCL4* upon WYMV infection. Three biological replicates were performed in this experiment. Data are means ± SD (*n* = 3). Asterisk indicates significant difference at *P* < 0.01 (one-way ANOVA).

Previous studies have shown that virus infection regulates the expression of RNA silencing-related genes in plants (Du et al., 2011; Conti et al., 2017; Sun et al., 2017). From our transcriptome analysis of wheat leaves (unpublished result), we have identified some RNA silencing-related genes including *AGO1*, *AGO2*, *AGO4*, *DCL2*, and *DCL4* based on their homology to the proteins encoded by other plant species (Supplementary Table S2). RT-qPCR analysis using the primers listed in Supplementary Table S1 showed that the transcription levels of wheat *AGO1*, *AGO2*, and *AGO4* in leaves and roots were significantly increased upon WYMV infection (**Figure 4**). Interestingly, wheat *DCL2* transcripts were upregulated in roots but downregulated in leaves and wheat *DCL4* transcripts were downregulated in roots but not in leaves upon WYMV infection (**Figure 4**). This result shows that WYMV infection affects the expression of *DCL* genes differently in leaves and roots. Studies using *A. thaliana* indicate that DCL4 and DCL2 generate 21- and 22-nt vsiRNAs, respectively, in a hierarchical manner (Deleris et al., 2006; Diaz-Pendon et al., 2007). Interestingly, in WYMV-infected wheat plants, upregulation of *DCL2* transcripts and downregulation of *DCL4* transcripts in roots coincided with a slightly higher proportion of 22- to 21-nt vsiRNAs relative to that in leaves (**Figures 1C,D**). The percentages of 22- and 21-nt vsiRNAs in roots were 22.5 ± 4.16 and 57.8 ± 3.04%, while they were 16.3 ± 2.42 and 65.1 ± 2.30% in leaves. Thus, WYMV infection in roots may affect the biogenesis of vsiRNAs by regulating transcript expression of *DCL* genes.

## Conclusion

In the present study, deep sequencing was used to characterize and compare the profiles of WYMV vsiRNA derived from leaves and roots of infected wheat plants. vsiRNAs in leaves and roots shared some similar characteristics in length distribution and polarity, while differed in abundance, hotspots distribution along viral genome and nucleotide bias at 5′ terminal. In addition, the expression of *DCL* genes (*DCL2* and *DCL4*) was differently regulated between leaves and roots upon WYMV infection. Overall, our results suggest divergent operation of RNA silencing defense against soil-borne virus invasion in roots. Further studies are necessary to investigate how WYMV infection regulates the expression of RNA silencing-associated genes and whether the differential regulation of *DCL2* and *DCL4* transcripts in roots affects the general activities of those genes and their preferential cleavage sites.

## Author Contributions

JC and JL conceived and designed the experiments. LL, YX, YZ, LH, and ZS performed the experiments. JL, IBA, GH, YC, FY, JY, and XX analyzed data. JC, JL, LL, and IBA wrote the manuscript.

## Conflict of Interest Statement

The authors declare that the research was conducted in the absence of any commercial or financial relationships that could be construed as a potential conflict of interest.

## Footnotes

1 http://hannonlab.cshl.edu/fastx_toolkit/

2 http://bowtie-bio.sourceforge.net

3 http://rna.tbi.univie.ac.at/cgi-bin/RNAWebSuite/RNAfold.cgi

4 http://weblogo.berkeley.edu/logo.cgi

## Supplementary Material

**FIGURE S1 |** Tissue-specific and common WYMV vsiRNAs (21 and 22 nt) based on unique reads. Three vsiRNA libraries (replicates) were mixed, and unique 21- and 22-nt vsiRNAs were extracted.

**FIGURE S2 |** Sequence logo analysis of WYMV siRNAs. 21- and 22-nt WYMV siRNAs corresponding to RNA2 were separately analyzed, and the 4 nt proximal to the 5′ and 3′ ends of the siRNAs in the viral genome sequence were included in the analysis. The overall height of the stack indicates the sequence conservation at that position, while the height of characters within the stack indicates the relative frequency of the nucleotide at that position.

**FILE S1 |** Viral small interfering RNA sequences in wheat leaves and roots.
