# Corrigendum: Differential Characteristics of Viral siRNAs Between Leaves and Roots of Wheat Plants Naturally Infected with Wheat Yellow Mosaic Virus, a Soil-Borne Virus

**DOI:** 10.3389/fmicb.2018.01477

**Published:** 2018-07-03

**Authors:** Linying Li, Ida Bagus Andika, Yu Xu, Yan Zhang, Xiangqi Xin, Lifeng Hu, Zongtao Sun, Gaojie Hong, Yang Chen, Fei Yan, Jian Yang, Junmin Li, Jianping Chen

**Affiliations:** ^1^College of Plant Protection, Nanjing Agricultural University, Nanjing, China; ^2^The State Key Laboratory Breeding Base for Sustainable Control of Pest and Disease, Zhejiang Academy of Agricultural Sciences, Hangzhou, China; ^3^Key Laboratory of Biotechnology in Plant Protection of Ministry of Agriculture of China and Zhejiang Province, Institute of Virology and Biotechnology, Zhejiang Academy of Agricultural Sciences, Hangzhou, China; ^4^Group of Plant-Microbe Interactions, Institute of Plant Science and Resources, Okayama University, Kurashiki, Japan; ^5^Institute of Plant Protection, Shandong Academy of Agricultural Sciences, Jinan, China

**Keywords:** soil-borne plant viruses, wheat yellow mosaic virus, viral small interfering RNA, antiviral RNA silencing, deep sequencing

In the published article, there was an error regarding the affiliations for Junmin Li and Jianping Chen. As well as having affiliation (2) and (3), they should also have (1).

There was an error regarding the affiliations for Yan Zhang and Lifeng Hu. As well as having affiliation (2), they should also have (3).

Additionally, in section Results and Discussion, in the title of sub-section: A/U Bias at the 5′-Terminal Nucleotide of vsiRNAs Was Lower in Leaves Than Roots, the word “Lower” should be changed to “Higher”. The sub-section title has been changed to read: A/U Bias at the 5′-Terminal Nucleotide of vsiRNAsWas Higher in Leaves Than Roots.

The authors apologize for these errors and state that they do not change the scientific conclusions of the article in any way.

The original article has been updated.

## Conflict of interest statement

The authors declare that the research was conducted in the absence of any commercial or financial relationships that could be construed as a potential conflict of interest.

