# Supplementary figures and images for: Differential Characteristics of Viral siRNAs between Leaves and Roots of Wheat Plants Naturally Infected with Wheat Yellow Mosaic Virus, a Soil-Borne Virus
Source: Front Microbiol. 2017 Sep 20;8:1802. doi: 10.3389/fmicb.2017.01802 (PMC5611437; doi:10.3389/fmicb.2017.01802)

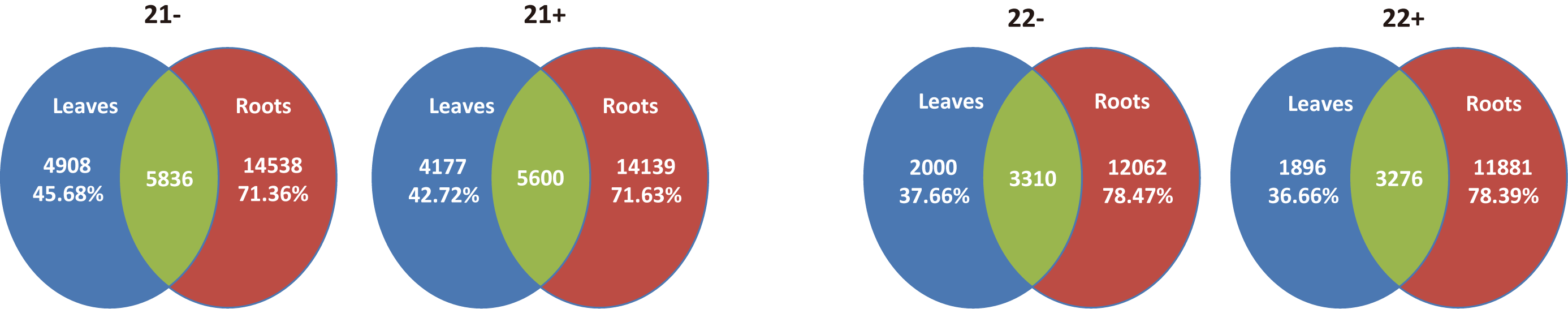

Supplement: Supplementary file 1 [file Image_1.TIF]

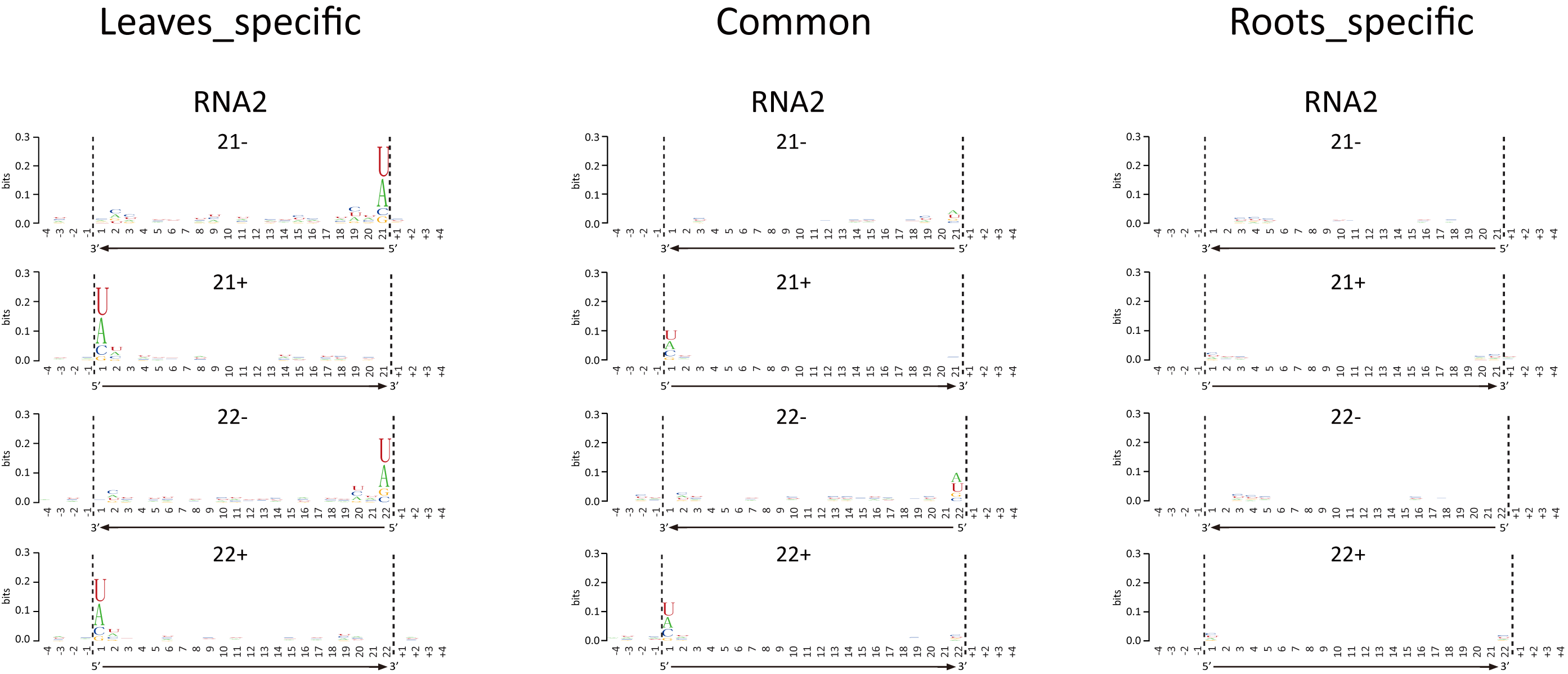

Supplement: Supplementary file 2 [file Image_2.TIF]
