# Supplementary material for: Differential Characteristics of Viral siRNAs between Leaves and Roots of Wheat Plants Naturally Infected with Wheat Yellow Mosaic Virus, a Soil-Borne Virus
Source: Front Microbiol. 2017 Sep 20;8:1802. doi: 10.3389/fmicb.2017.01802 (PMC5611437; doi:10.3389/fmicb.2017.01802)
Supplement: Supplementary file 4 [file Table_1.DOCX]

Table S1. RT-qPCR primer sequences used in this study.

| Primer | Primer sequence (5'-3') |
| --- | --- |
| WYMV-CP-F | GGACCTCACAGCAACCAAGG |
| WYMV-CP-R | CCAGAGCGATGGAGTTGTCG |
| TaU-F | ATCCAGGACAAGGAGGGCA |
| TaU-R | CGGAGACGGAGCACCAAG |
| TaAGO1-F | ACGGTGGTAGGGTCAGTCAC |
| TaAGO1-R | GGTTCAGGTGCAAAGTCCAT |
| TaAGO2-F | CCGTGTCAGAGTTCGTTCAA |
| TaAGO2-R | TAGAAGCCTCACGCACAATG |
| TaAGO4-F | CAGTCTGCCATCCAAGGAAT |
| TaAGO4-R | CCGCAGCAAAGTGTATCTCA |
| TaDCL2-F | CCATTTGCCACTACGAGGAT |
| TaDCL2-R | GCTTTTCTTGCAACCTGTCC |
| TaDCL4-F | CACTTCTGCACGCTAAGCAG |
| TaDCL4-R | AGGCAACTCCACAAAGTGCT |
