# Supplementary material for: Differential Characteristics of Viral siRNAs between Leaves and Roots of Wheat Plants Naturally Infected with Wheat Yellow Mosaic Virus, a Soil-Borne Virus
Source: Front Microbiol. 2017 Sep 20;8:1802. doi: 10.3389/fmicb.2017.01802 (PMC5611437; doi:10.3389/fmicb.2017.01802)
Supplement: Supplementary file 5 [file Table_2.docx]

Table S2. Wheat *ago* and *dcl* genes derived from transcriptome, based on their homology to genes of other plants

| Genes | Accession | Homology accession (plant) | Blast Identities |
| --- | --- | --- | --- |
| *Ago1* | KY794779 | XP_010240271.1 (*Brachypodium distachyon*) | 637/670(95%) |
| *Ago2* | KY794780 | BAJ95476.1 (*Hordeum vulgare*) | 808/845(96%) |
| *Ago4* | KY794781 | XP_010230772.1 (*Brachypodium distachyon*) | 834/927(90%) |
| *Dcl2* | KY794782 | XP_014752438.1 (*Brachypodium distachyon*) | 379/491(77%) |
| *Dcl4* | KY794783 | XP_010240123.1 (*Brachypodium distachyon*) | 1152/1394(83%) |
